# Multiple-membership multiple-classification models for social network and group dependences

**DOI:** 10.1111/rssa.12021

**Published:** 2014-08-09

**Authors:** Mark Tranmer, David Steel, William J Browne

**Affiliations:** University of ManchesterUK; University of WollongongAustralia; University of BristolUK

**Keywords:** Auto-correlation, Linear regression, Multilevel models, Social networks

## Abstract

The social network literature on network dependences has largely ignored other sources of dependence, such as the school that a student attends, or the area in which an individual lives. The multilevel modelling literature on school and area dependences has, in turn, largely ignored social networks. To bridge this divide, a multiple-membership multiple-classification modelling approach for jointly investigating social network and group dependences is presented. This allows social network and group dependences on individual responses to be investigated and compared. The approach is used to analyse a subsample of the Adolescent Health Study data set from the USA, where the response variable of interest is individual level educational attainment, and the three individual level covariates are sex, ethnic group and age. Individual, network, school and area dependences are accounted for in the analysis. The network dependences can be accounted for by including the network as a classification in the model, using various network configurations, such as ego-nets and cliques. The results suggest that ignoring the network affects the estimates of variation for the classifications that are included in the random part of the model (school, area and individual), as well as having some influence on the point estimates and standard errors of the estimates of regression coefficients for covariates in the fixed part of the model. From a substantive perspective, this approach provides a flexible and practical way of investigating variation in an individual level response due to social network dependences, and estimating the share of variation of an individual response for network, school and area classifications.

## 1. Introduction

Many social populations have a multilevel structure, such as individuals in households and areas, students in schools, workers in organizations or observations made on individuals at different time points. Multilevel models have been developed to analyse data collected from these populations (see, for example Snijders and Bosker (2012) and Goldstein (2003)), and to allow relationships between variables to be investigated at a particular level or classification, often the individual level, while taking into account effects operating at other classifications of the population structure, e.g. schools or areas. In some situations, the aim of the analysis is to assess whether an individual level relationship differs from one group to another. For example, is the relationship between prior and current examination performance stronger in some schools than others? Multilevel modelling can also be used as a model-based approach for analysing data that have been collected from a multistage sample, when the target of inference is the individual level relationship between a set of variables. In this approach the primary sampling unit is included as a level, and the population structure may be regarded as a nuisance to be taken into account in the analysis of an individual level relationship, so that regression coefficients are estimated efficiently, and appropriate standard error estimates are calculated. However, more often in multilevel analysis the population structure is of direct substantive interest, and the aim of the analysis may be twofold: to estimate an individual level relationship given the complex population structure, and to measure the nature and extent of variations in variables of interest at the various levels of this population structure.

Numerous studies have been carried out to investigate household level, school level or area level variations in individual level responses for social, educational and health outcome variables by using multilevel models. However, social networks have been largely ignored in the multilevel modelling literature as an *additional* source of dependence. Network auto-correlation models have been developed for social network dependences in the social network literature, adapted from models that were originally developed for spatial dependences (Cliff and Ord, 1975; Ord, 1975). For network auto-correlation models it is typically assumed that the response variable is continuous, though there have been some very recent developments to this model for a dichotomous response (Koskinen and Stenberg, 2012). Although network auto-correlation models take the social network dependences into account, they essentially ignore other levels of the population structure unless these are added to the model as fixed effects, which can be problematic when there are many groups. Moreover, network auto-correlation models do not allow an easy assessment of the relative share of variation in an individual response at the individual, network and group levels.

This paper illustrates some new approaches for assessing social network dependences in an individual response variable—academic performance—while including other group dependences such as the school or area to which an individual belongs, by applying multiple-membership multiple-classification (MMMC) models (Hill and Goldstein, 1998; Browne *et al*., 2001) to the Adolescent Health Study data set (Harris *et al*., 2009). The main aim of the analysis is twofold: to estimate an overall individual level relationship between a set of variables, and to measure the nature and extent of variations in individual level responses at various levels of the population structure. There are two other aims of the analysis: an assessment of the consequences, in terms of model parameter estimation and subsequent statistical inference, of ignoring different features of the population structure in the analysis, and a comparison of multiple-membership (MM) models with existing models for network auto-correlation.

An empirical study has been carried out using a subsample of the Adolescent Health Study data for one state in the USA, focusing on an individual level response variable: academic performance, which is related to three individual level covariates: gender, ethnic group and age. Friendship networks are available for the individuals in the Adolescent Health Study data. The levels of the population structure that are studied alongside the social network are area (defined by US county) and school, as well as the individual level. The data have been analysed before when investigating network effects in education, from a different perspective from that of the present study. For example, Calvó-armengol *et al*. (2008) used the data to study peer effects and social networks in education. They were particularly interested in an individual's position in their peer (friendship) network and used a peer effects model, based on a variation of the Anselin (1988) spatial error model, in their investigation. They found that, after controlling for observable individual characteristics and unobservable network-specific factors, the individuals' position in a network, based on a centrality measure, is a key determinant of their academic performance.

In the MMMC approach for social networks, individuals are members of ego-nets and cliques. Cliques are subnetworks of a particular size where every individual is directly connected to one another. For example, in a clique of size 3, all three individuals are connected. A clique of size 2 is more often referred to as a dyad, in which the pair of individuals are connected. An ‘ego-net’ consists of a particular individual in the network and all the other individuals in the network to which they are directly connected. Each individual may belong to more than one of these groups, or in some cases not be a member of any groups—e.g. population network isolates, or members of the sampled network with very few connections to other sampled individuals. Members of the network with few connections are more likely to occur in sampled networks, because the friends whom sampled individuals nominate may be out of sample. Other group dependences, such as those at the school or area level, are added as additional crossed levels in the MMMC model, which is an extension of the multilevel model.

Where possible in this paper, the results of the MMMC approaches are compared with existing approaches for taking into account social network dependences: in particular, network auto-correlation models. In addition, comparisons are made with a single-level regression model, which ignores all higher level features of the population structure. It is of interest to assess whether such a naive statistical modelling approach would lead to different substantive conclusions when compared with those models that account for population structure, as well as assessing the consequences for statistical estimation of ignoring levels of the population structure above the individual.

The remainder of the paper is organized as follows. In Section 2, a description of the data structure and preparation is given, together with some descriptive results. In Section 3 a discussion of the modelling approaches is given, including a brief review of network auto-correlation models, and a definition of the MMMC models for social network and group dependences. In Section 4, the results of analysing various models are given. Finally, a discussion is given and conclusions are drawn in Section 5.

## 2. Data description and preparation

The data are from a restricted access Adolescent Health Study data set for wave I (Harris *et al*., 2009) and were analysed in a secure data environment. The Adolescent Health Study is a longitudinal sample of school students in grades 7–12 (typically aged 11–16 years) in the USA, which began around 1995. Information that was collected includes a range of health outcome variables, academic performance and individual (i.e. student level) characteristics, as well as school and geographical details, and friendship networks. A subset of the data was used for ‘State 7’, which is a state of the USA which comprises 968 valid sampled cases for individuals in 10 schools, and in 13 areas at the county scale. The dependent variable is a measure of academic performance, which was generated by converting the grades from four subjects (graded A–D) into four five-point scales (1, D or less, 2, C, 3, B, and 4, A) and then summing them. These scores were then standardized to have mean 0 and standard deviation 1 and are called ‘ztotscore’ in the tables.

The friendship networks are potentially available for all students in the sample. Each individual is asked to name up to five male and five female friends. These friends may also be in the sample, or friends can be named who are out of sample. The networks are available in the Adolescent Health Study data as adjacency (node) lists; for each individual, the adjacency list is simply a list of names of their friends (in anonymized form). Directed binary adjacency matrices were extracted from these lists by using R (R Core Team, 2012), via a routine for converting adjacency lists to adjacency matrices. For a sample of size *n*, the binary adjacency matrix **D** has dimensions *n*×*n*. An element 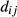 in the matrix **D** takes the value 1 if *i* nominates *j* as a friend and 0 otherwise. Individual *j* may reciprocate by nominating *i* as a friend, or may not, so the directed adjacency matrix is not usually symmetric. Diagonal elements 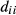, which would indicate self-friendship nominations, are assumed to be 0. These adjacency matrices were restricted to other individuals in the sample, and also in State 7, resulting in sample network isolates. This does not present problems in the modelling approaches that are discussed later, but it is worth noting that the structure of a sampled network is different from a population network; a sample network is likely to be considerably more sparse and less structured than the corresponding population network. As well as obtaining the directed adjacency matrix **D**, a symmetrized (undirected) version of this matrix, 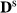, was obtained and was used in the clique set analyses below. For a symmetrized matrix 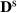, if *i* nominates *j* as a friend, if *j* nominates *i* as a friend, or if both nominate each other as friends, the element 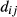 is given a value of 1. Thus, the symmetrized matrix is, by definition, symmetric, and it tends to have much higher ‘density’ (i.e. will have more of the possible network connections present) than the directed adjacency matrix **D** from which 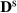 was derived.

Where the network level was considered, the MMMC models that are applied in Section 4 involved clique memberships for undirected cliques of minimum size 2 (clique-2) and of minimum size 3 (clique-3), as well as ego-net membership; an ego-net is an individual's direct network of friends, where the individual is defined as the *ego* and their friends are defined as their *alters*. When clique-2 and clique-3 definitions are used together in the models, the clique-2 level represents dyads only (a dyad is a pair of directly connected individuals), and the clique-3 level represents cliques of size at least 3 (for a clique of size 3, all three members of the clique are directly connected to one another). In theory, cliques of minimum size 4, or other network configurations, could be specified in this approach, but there are too few of these to include in the models for these sample data. The clique sets for a minimum clique size of 2 (clique-2) and minimum size of 3 (clique-3) were obtained by using UCINET 6 (Borgatti *et al*., 2002). The program implements the Bron and Kerbosch (1973) algorithm to find all Luce and Perry (1949) cliques of a specified minimum size. The ego-nets for each individual were extracted from the adjacency matrix; the ego-net for each individual is the corresponding row of the adjacency matrix. Once the ego-nets and clique sets had been obtained, weight matrices were produced for each individual. These weights work as follows. If an individual is a member of four clique-3s, their weights for each of these clique-3s will be 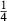. Where ego-nets are used in the models a similar approach is used: if an individual (ego) had three friends in the sample (alters), each of these alters would each be given a weight of 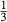 in the weight matrix.

### 2.1. Descriptive statistics

#### 2.1.1. Attributes

Three covariates (attributes) were included in the models: ethnicity, which is coded as a binary indicator about whether the student is black American or not, gender and age. The first two covariates are categorical, and the third is continuous and is centred in the modelling results that appear in Section 4. The total sample size is 968, comprising 510 (52%) females and 161 (17%) black Americans. The average age in the sample is 14.76 with a minimum of 10 and a maximum of 19.

#### 2.1.2. Social networks

Each row of the directed network adjacency matrix represents the ego-net of an individual, in terms of whom they nominate as friends. Thus, if individual *i*, represented by row *i* of the matrix **D**, nominates four friends, then the four off-diagonal elements in row *i* of **D** that represent these friends will take the value 1 and the remaining *n*−4 elements will take the value 0. For an isolate in the sample data, the entire row for that individual will have elements of value 0. The ego-net size ranges from zero (in the sample) (317 people, 39%) to nine people in the sample (one person, 0.1%). Among those with a non-zero ego-net the mean ego-net size is 1.80 with a standard deviation of 1.22. The clique analyses are based on the undirected adjacency matrix. 34% (333 students) do not belong to any clique-2s. The mean number of clique-2s that students belong to, overall, is 1.39. For those students who belong to at least one clique, the mean number of clique-2s that they belong to is 2.11. One student is a member of 12 clique-2s, which is the maximum of this distribution. Fewer students are members of clique-3s; 805 students (83%) do not belong to any clique-3s. The mean number of cliques that each student belongs to is 0.32. For those students who belong to at least one clique, the mean number of clique-2s that they belong to is 1.91. The maximum number of clique-3s that any student belongs to is 11, for one student in the data set.

#### 2.1.3. Groups

The groups that are considered in this study are schools and areas. There is a strong relationship between schools and areas: for six of the schools all the students are from one area in each school; for each of the four other schools, the majority of students are from one area, with just one or two students from other areas. The distribution of people in areas, where the areas here are counties, is uneven. Some areas have a large sample size, whereas some other areas contain only one observation. There are six areas with sample sizes greater than 50. The sample is much more evenly distributed in the 10 schools that are included in State 7, because the sample design is a school-based study.

There are also two other finer scales of geography in the Adolescent Health Study data set: ‘neighbourhood 1’ and ‘neighbourhood 2’, where the latter definition is the finest geographical scale, though both these neighbourhood definitions often include only one sample unit per area. Although substantively these geographical scales may be interesting to study, there were insufficient data to consider these areas further as classifications in the models, as it would be very difficult to disentangle individual and area level effects at these scales.

## 3. Models

### 3.1. Multiple-membership multiple-classification models

Social network and group dependences can be taken into account through a random-effects modelling approach via an extension to the multilevel model, known as the MMMC model, which is itself an extension of the MM model to include other group dependences. Before defining the full MMMC model for social network and group dependences, we first adapt the notation of Browne *et al*. (2001), equation 5, for the MM model with social network dependences only.

The MM model for social network dependences may be defined by one network subgroup (e.g. membership of ego-nets) as 
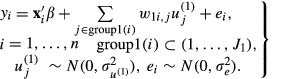
1

In model (1), 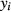 is an individual level response. In this model formulation this response is assumed to be continuous, 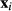 is a vector of fixed covariates and ***β*** is the vector of their regression coefficients. Here, group1(*i*) is the set of ego-nets to which *i* is a member. The term 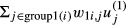 involves a set of 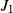 random effects 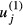, where 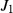 is the total number of ego-nets. The weight that is given to each individual for their ego-net membership is 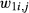. These weights sum to 1 for each individual. The random effects at the individual and network levels are assumed to be uncorrelated. In these models, the ego-net of each individual is represented only by their friends, so ego is not included in their own ego-net, although alternative weighting schemes could be used. Strictly speaking, model (1) is an MM model, rather than an MMMC model, because in this model there is only one classification.

The MMMC model for social network dependences may be defined by two network subgroups (e.g. membership of cliques of minimum size 2, and membership cliques of minimum size 3) as 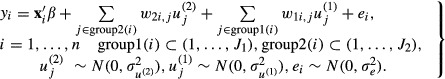
2

The term 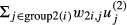 involves a set of 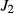 random effects 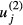, where 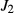 is the total number of network subgroups for the second network definition included in the model. The weight that is given to each individual for their membership of this second set of network subgroups is 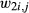, and these weights sum to 1 for each individual. Model (2) is an MMMC model, because now two classifications are included in the specification.

It is possible to put both clique-2s and clique-3s in the model as two sets of classifications. Although some clique-2s are nested within clique-3s, this is similar to the more familiar case of a three-level multilevel model, with pupils in classes in schools, where clearly the schools (which are analogous to clique-3s) contain the classes (analogous to clique-2s). For both the pupils in classes in schools case and the individuals in clique-2s in clique-3s case, the estimated variation is divided between the individual level and the two classifications.

Finally, models (1) and (2) may be further extended to allow for both social network and group dependences. Since group dependences have now been added to the models in the form of area and school classifications, these are MMMC models: both for the case where ego-nets are used for the social network definition, as well as for the case where the clique definitions are used. For example, in model (3) the model for social network dependences with two network subgroups, model (2), is extended to include two additional classifications: a geographical classification, as defined by county, with random effects 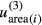 and a school classification, with random effects 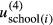. The area and the school are not assumed to be nested in this model formulation, although there is a strong correspondence of schools to areas in this data set: 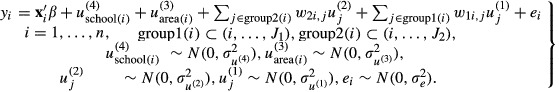
3

Here school(*i*) is the school to which student *i* belongs, and area(*i*) is the area in which student *i* lives. In some of the analyses of the consequences of ignoring a classification later in the paper, model (3) was also fitted without the network components included. Also, because there is a close correspondence of particular schools to particular areas, a slightly reduced version of model (3) was also fitted with just the school classification included, or just the area classification included. The results are summarized in Tables 1, 2 and 3 in Section 4.

**Table 1:** Null MMMC models^†^

*Parameter*	*ztotscore for the following levels:*							
	*School only*	*Area only*	*Network only*	*School + area*	*School + network*	*Area + network*	*School + area + network*	*Individual only*
Constant	0.04	0.03	−0.02	0.03	0.02	0.02	0.03	0.00
	(0.10)	(0.10)	(0.03)	(0.12)	(0.09)	(0.11)	(0.10)	(0.03)
School variance	0.075			0.059	0.073		0.052	
Area variance		0.070		0.036		0.070	0.036	
Network variance								
Clique-3			0.188		0.148	0.218	0.153	
Clique-2			0.139		0.139	0.040	0.139	
Individual	0.958	0.973	0.924	0.958	0.884	0.930	0.885	1.00
variance								
DIC	2714	2727	2736	2715	2700	2716	2701	2750

† Dependent variable, academic performance (ztotscore). Social networks are defined by undirected clique-2 and clique-3 membership.

**Table 2:** Main effects MMMC models with individual covariates^†^

*Parameter*	*ztotscore for the following levels:*							
	*School only*	*Area only*	*Network only*	*School + area*	*School + network*	*Area + network*	*School + area + network*	*Individual only*
Constant	−0.09	−0.07	−0.09	−0.08	−0.10	−0.09	−0.09	−0.08
	(0.07)	(0.09)	(0.05)	(0.09)	(0.07)	(0.09)	(0.08)	(0.05)
Black	−0.03	−0.04	−0.07	−0.03	−0.03	−0.04	−0.03	−0.06
	(0.09)	(0.09)	(0.09)	(0.09)	(0.09)	(0.09)	(0.09)	(0.08)
Female	0.17	0.17	0.17	0.17	0.17	0.17	0.17	0.17
	(0.06)	(0.06)	(0.06)	(0.06)	(0.06)	(0.06)	(0.06)	(0.06)
Age	−0.13	−0.12	−0.13	−0.12	−0.12	−0.12	−0.12	−0.13
	(0.02)	(0.02)	(0.02)	(0.02)	(0.02)	(0.02)	(0.02)	(0.02)
School variance	0.023			0.010	0.027		0.012	
Area variance		0.034		0.029		0.036	0.026	
Network variance								
Clique-3			0.106		0.110	0.106	0.102	
Clique-2			0.016		0.103	0.099	0.108	
Individual	0.930	0.927	0.922	0.927	0.875	0.875	0.871	0.941
variance								
DIC	2687	2682	2689	2683	2678	2674	2674	2692

† Dependent variable, academic performance (ztotscore). Social networks are defined by undirected clique-2 and clique-3 membership.

**Table 3:** Main effects MMMC models^†^

*Parameter*	*ztotscore for the following levels:*							
	*Network only*	*School + network*	*Area + network*	*School + area + network*	*Network only*	*School + network*	*Area + network*	*School + area + network*
Constant	−0.01	0.03	0.07	0.04	−0.09	−0.09	−0.07	−0.08
	(0.03)	(0.09)	(0.06)	(0.1)	(0.04)	(0.07)	(0.09)	(0.09)
Black					−0.06	−0.03	−0.04	−0.03
					(0.09)	(0.09)	(0.09)	(0.08)
Female					0.17	0.16	0.17	0.17
					(0.06)	(0.06)	(0.06)	(0.06)
Age					−0.13	−0.12	−0.12	−0.12
					(0.02)	(0.02)	(0.02)	(0.02)
School variance		0.08		0.06		0.03		0.01
Area variance			0.07	0.03			0.04	0.03
Network variance								
Ego-net	0.17	0.20	0.15	0.19	0.15	0.15	0.15	0.15
Individual level	0.926	0.870	0.902	0.873	0.873	0.861	0.856	0.862
variance								
DIC	2737	2697	2713	2697	2681	2674	2669	2672

† Dependent variable, ztotscore. Social networks are defined by ego-net membership.

An alternative way of accounting for the group dependences is to extend model (1) or (2) to include fixed indicator variables for the groups, and this may be appropriate where the number of groups is small and all groups are included in the sample. An example of this approach, where there are fixed indicator variables for schools, rather than adding school as a classification (and where areas are not included in the model), can be found in Table4 in Section 4. Technically, this modelling approach is an MM, rather than an MMMC, model, because the schools are included in the fixed part of this model via dummy variables, rather than in the random part of the model as a classification; hence this model is referred to as an MM model in the discussion of the results in Table4.

**Table 4:** MMMC and network disturbance models with school indicators as fixed covariates^†^

*Parameter*	*ztotscore for the following levels:*			
	*Constant + school indicators*		*Full model*	
	*MMMC*	*Network disturbance*	*MMMC*	*Network disturbance*
Constant	−0.004 (0.110)	0.001 (0.104)	0.030 (0.115)	0.034 (0.110)
School 2	0.259 (0.153)	0.235 (0.152)	0.076 (0.160)	0.043 (0.159)
School 3	−0.196 (0.128)	−0.205 (0.129)	−0.174 (0.134)	−0.180 (0.128)
School 4	−0.107 (0.131)	−0.136 (0.128)	−0.112 (0.130)	−0.131 (0.126)
School 5	−0.327 (0.151)	−0.342 (0.156)	−0.296 (0.156)	−0.307 (0.154)
School 6	0.344 (0.165)	0.322 (0.164)	−0.012 (0.178)	−0.044 (0.178)
School 7	0.138 (0.156)	0.123 (0.158)	−0.185 (0.173)	−0.207 (0.170)
School 8	0.407 (0.167)	0.388 (0.167)	0.050 (0.185)	0.022 (0.180)
School 9	0.258 (0.151)	0.251 (0.152)	−0.083 (0.167)	−0.099 (0.166)
School 10	−0.327 (0.173)	−0.327 (0.173)	−0.612 (0.189)	−0.626 (0.186)
Black			0.006 (0.092)	0.015 (0.093)
Female			0.165 (0.062)	0.173 (0.062)
Age			−0.119 (0.025)	−0.125 (0.025)
Ego-net variance	0.193		0.165	
Individual variance	0.872		0.855	
DIC	2698		2674	
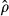		0.107 (0.041)		0.111 (0.041)
AIC		2711		2675
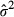		0.917		0.883

† The full model also includes all individual level covariates. Dependent variable, academic performance (ztotscore); reference school, school 1.

Models (1), (2) and (3) can all be estimated in the software MLwiN (Rasbash *et al*., 2009). The model parameters are estimated via Markov chain Monte Carlo sampling (Browne, 2009). Before fitting the models, data preparation to identify the groups in the networks, such as ego-nets, or cliques, and the corresponding membership weights, as discussed above in Section 2, must be undertaken. Model (3) was fitted to the Adolescent Health Study data in the form as specified above with area, school, network and individual levels all defined, and such a model was run as a null model (no covariates) and as a model with fixed covariates included. In this model, the two levels of network dependences were clique-2s and clique-3s, based on the undirected adjacency matrix. A slightly reduced form of model (3) was also fitted, with just one level of network dependences: the ego-nets of each individual, as derived from the directed adjacency matrix. The results are discussed in the next section.

Fitting these models allows the two main aims of the analysis to be achieved: firstly, to estimate the individual level relationship between the individual level outcome variable, academic performance, and the three individual level covariates gender, ethnic group and age, having taken the clustering of academic performance in networks, schools and areas into account, and, secondly, to investigate the extent of variation in academic performance at the individual, network, school and area levels, both before and after the inclusion of individual level covariates. In addition, reduced versions of this model were also fitted, including a single-level regression model, i.e. a model that ignores school, area and network dependences, as well as models that ignore some of the levels above the individual. This achieves one of the other aims of this paper; to investigate the consequences of ignoring particular features of the population structure in statistical analysis. The results are given in Section 4.

### 3.2. Network auto-correlation models

Other models exist for social network dependences, and these have been applied in the social networks literature; in particular, *network auto-correlation models*. See Leenders (2002) for a review. One such network auto-correlation model is the *network effects* model, which is also known in the geographical literature as the *spatial effects* model (Doreian, 1980), and defined as 
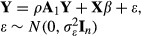
4 where **Y** is an *n*×1 vector of response variables and 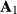 is an *n*×*n* matrix of weights to reflect the connections for the *n* individuals. The definition of 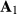 will affect the estimated model parameters (Leenders, 2002). One such definition, as used here, is to derive 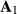 from the row-standardized directed adjacency matrix, so that this is the same weight information as used in the MM models based on ego-nets (see the definition above). The auto-correlation parameter *ρ* measures the strength of association between an individual's response and the responses to which the individual is connected. The same value of *ρ* is assumed for all of these connections. **X** is an *n*×*p* matrix of fixed covariates, with associated regression coefficients *β*, and *ɛ* is a vector of error terms. If there are no connections between individuals, or if *ρ* is 0, model (4) reduces to an ordinary least squares regression equation.

A variation of this model the *network disturbances* model, which is also known in the geographical literature as the *spatial disturbances* model (Doreian, 1980), is defined as 
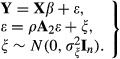
5

In model (5) the parameters in common with model (4) are defined identically. The matrix 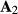 is an *n*×*n* weight matrix to reflect the connections for the *n* individuals, which could be identical to 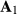. Again, if there are no connections between individuals, or if *ρ* is 0, model (5) reduces to an ordinary least squares regression equation. Finally, it is possible to formulate a combined model based on models (4) and (5), so that network auto-correlation is jointly modelled through the response and error terms, and where the matrices 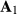 and 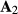 may be the same, or different. Models (4) and (5), and the combined model, may be estimated by using the sna and spdep packages in R (see, respectively, Butts (2008) and Bivand (2013)). In the literature, the models tend to perform similarly in terms of statistical measures (Doreian, 1980; Leenders, 2002). The decision to fit the network effects model, network disturbances model or some combination of the two should therefore be made on substantive grounds.

### 3.3. Different modelling approaches

As well as the social network dependences, other group dependences may also be present. For example, in the Adolescent Health Study data, there are geographical groups at various scales (neighbourhoods, and counties), as well as schools. In the fixed effects approaches of models (4) and (5), it is possible also to add the groups as fixed effects via a set of indicator (dummy) variables. This approach is often appropriate when there only a few groups. Some of these models were estimated below with schools as groups. This enabled a comparison of the network disturbance model and MM models to be made for an example that included network and group dependences (see Section 4 for a discussion). When modelling the cliques in the MM models, it would be possible to include just one term, e.g. just for clique-3s without clique 2s, and this may be appropriate if this corresponded to a particular substantive theory about cliques of size 3 or more with respect to the individual level responses.

The decision to fit network auto-correlation models or MMMC models when other groups are present is likely to depend on two considerations: one substantive; one practical. Firstly, from a substantive perspective, if interest focuses on estimating the nature and extent of variation in individual responses at the network classifications compared with other group classifications, then the MMMC model may be preferable because variance components are explicitly included as model parameters and it is possible to model other group classifications alongside the networks as random effects and to compare the extent of variation for these different classifications. However, if the substantive focus of the analysis is on estimating the individual level relationship and the networks are regarded as a nuisance, then the network auto-correlation model may be preferable. Moreover, the feedback correlations *ρ* that are obtained from these models may have a substantive interpretation in some contexts. Secondly, from a practical perspective, if the number of groups such as schools or areas is large, and/or if the sample is unevenly distributed within the groups, the MMMC modelling approach may be more statistically efficient. Also it is relatively straightforward to define and fit the MMMC model for a binary response, whereas it is not yet straightforward to fit the network auto-correlation model for a binary response, although such models have recently been fitted by Koskinen and Stenberg (2012). In the next section, the MMMC and network auto-correlation models are compared, using the Adolescent Health Study data for the academic performance response variable, which is continuous.

## 4. Results

MMMC models for network and group dependences were fitted, with standardized academic score ztotscore as the dependent variable. In Tables1 and 2 the network was defined through the clique-2 and clique-3 membership, on the basis of the undirected adjacency matrices. All MMMC models presented in this paper were estimated via a Markov chain Monte Carlo algorithm by using default flat priors for the fixed effects and a chain of 20000 samples, implemented in MLwiN. In all models, standard diffuse (gamma) priors were assumed for the variance parameters.

Table1 shows null models with only a constant term in the fixed part, and Table2 adds the three individual level fixed covariates. Because both clique membership terms are included in the models, clique-2 represents dyad membership only, and clique-3 represents individuals who are members of cliques of minimum size 3.

Network, school and area levels were included in the complete models (the penultimate columns of Tables1 and 2) to meet the two main aims of this study:

1. to estimate the individual level relationship between academic performance and the individual level covariates, having taken into account the population structure above the individual level, and
2. to investigate the nature and extent of variations in individual level responses at the individual and group levels.

In Table1 for the null models, the best fitting models according to the deviance information criterion DIC involved the network and the school classification above the individual level (DIC = 2700). For the model that included schools, areas and networks there was little difference in the goodness of fit (DIC = 2701) when compared with the model that includes only networks and school classifications, probably reflecting the overlap of schools and areas in this example. The areas may not perform quite as well as a level when schools are not included because of the uneven sample distribution in areas (DIC = 2716). The final column of Table1 shows the results for the null model with a DIC of 2750, suggesting a much worse model fit when no population structure above the individual level is accounted for. These goodness-of-fit results suggest that the population structure above the individual level, including the social networks, should not be ignored in statistical analysis when studying variations in academic performance.

The penultimate column of Table1 shows the results for the model that includes networks, schools and areas. It can be seen that most of the variation is between individuals but considerable variation is also associated with networks for both clique definitions, suggesting clustering of academic performance within dyads (clique-2s) and also within more structured parts of the network (clique-3s). There is more variation between schools than between areas (variance components 0.052 and 0.036 respectively), although as discussed earlier there is considerable overlap of schools and areas in this sample. In the two models that fit only either the school or the area as the group and also include network variance components, the network variance components still appear to be important.

A key feature of the multilevel modelling approach is to assess the relative share of variation at the different levels specified in the models—schools, areas, networks and individuals. Considering the MMMC modelling extension given by equation (3), each student can belong to only one area and to one school (equivalent to a weight of 1 for the school and the area that they belong to) but can be a multiple member of network subgroups such as ego-nets or cliques with differential weights per individual. The network level random effects in the MMMC model, model (1), have weights 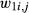 for each individual, and for model (2) also involve a second set of weights 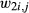 for each individual. Therefore, when considering the contribution to the overall variance, the contribution of the network level for each individual is 
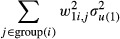
 for model (1) (and similar contributions in model (2)). These weights will potentially be different for each individual and will be 0 for an individual who is not a member of any particular network subgroups to which the weights correspond. In the models we generally assume equal weights for each group and that these weights sum to 1 so that the above formula can be simplified to 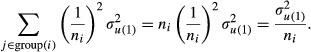


In other words the importance of the network effects is inversely proportional to the number of network subgroups that the individual belongs to, 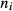, which makes sense; as one belongs to more networks the effect of any particular network will reduce. Therefore the network variance components cannot be directly compared with the components at the other levels; it is evident from the tables for the null models that if the area, school, individual and network variance components are added together these result in a total variance that is much greater than 1, which does not correspond to the variance of a standardized variable (which should have a value of 1). One possibility is to consider the effect of the various classifications for an average individual, by averaging the variance for each individual across the data set to obtain an average variance component for the network effects: 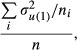 where 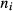 is the number of networks for individual *i*, and *n* is the total number of individuals. Using this approach and for the Adolescent Health Study data, the average weights were calculated as follows: mean ego-net weight, 0.453; mean clique-2 weight, 0.420; mean clique-3 weight, 0.119. If the variance components for the respective network subgroups are multiplied through by these values, the total variance now sums to approximately 1 for the null models, as expected, and a more meaningful comparison can be made regarding the relative share of variation at the individual, area, school and network levels. This approach makes the MMMC modelling approach useful for obtaining a descriptive measure of the variation in the response at each of the levels that are included in the model. For example for the ‘School+network’ model in Table1, approximately 7.3% of variation in academic performance is at the school level, approximately 1.7% at the clique-3 level, approximately 5.8% at the clique-2 level and approximately 88.4% of variation is at the individual level. These percentages do not add to exactly 100% possibly because of cluster imbalance and the prior distributions that were used in the Markov chain Monte Carlo estimation, but they give a good indication of the sources of variation in academic performance at the school, network and individual levels.

The weights that are used throughout the paper for the modelling of the network classifications, for both ego-nets and cliques, are inversely proportional to the total number of friends, or cliques, to which each individual belongs, and the weights are equal for each friend. Such a weighting scheme seems intuitively sensible and has parallels with other models such as the network disturbances model but also has substantive implications. In particular, people with more friends in the sample have smaller weights for each friend than those people with fewer friends. Although not the main aim of the paper, it is of interest to investigate, empirically, the effects of changing these weighting schemes.

A sensitivity analysis was carried out with different weights by using three schemes: firstly, the scheme that was used for all the models throughout the paper, where weights are inversely proportional to the number of friends; secondly, weights of 1 for any friend, so that the weights will add up to *n* rather than to 1 for each individual with at least one friend; finally, weights of 1/√*n*, which are inversely proportional to a function of *n* but will not add up to 1 for every individual. The weights for people with more friends will be reduced less for this third scheme. These three weighting schemes were compared for null models with individual, network and school classifications, where networks were defined both by clique-2s and clique-3s, and by ego-nets (the results are not shown). The estimates for the school variance component changed very little, whichever weighting scheme was used, but the individual level (residual) variance is slightly affected by the choice of weights in the network component. This is to be expected as the amount of residual variation explained by the network will depend on the weighting scheme.

In Table1 features of the population structure were removed from the models to assess the consequences of ignoring one or more levels of population structure in this example. If just schools or just areas are modelled above the individual, the individual variance component increases compared with the cases where the network level is also included, but the school or area level components do not change much. This is in contrast with the findings of Tranmer and Steel (2001) who found that ignoring a geographical level between the individual level and a more aggregated area level affected both estimates in an example based on UK census data. This reflects the fact that the levels were hierarchical in the case of census data for areas. In the case of the Adolescent Health Study data, the population has a cross-classified structure.

Looking now at the model that adds the three individual level covariates in Table2, the pattern is fairly similar to that described for Table1 in terms of model goodness of fit. The penultimate column of Table2 gives the model results with all three levels above the individual (school, area and network) included. For the individual level relationship between academic score and the three covariates there is little change in the coefficients, and only a minor reduction in their standard errors, although the coefficient for ‘Black’ changes in its point estimate (though it is non-significant in both cases) and the standard error increases a little for the model that includes population structure above the individual level.

In terms of the share of the variation at the network, area and school level (the penultimate column of Table2) the pattern is similar to that described above for the null model (Table1). In all models, the coefficient estimates for female and age are found to be statistically significant and, conditional on the addition of the covariates to the model, the school and area level variance components reduce. The clique-3 variance components are also reduced by the inclusion of covariates and, interestingly, in the model with all features of the population structure included (the penultimate column) there is now a slightly larger variance for the clique-2 level than the clique-3 level, though both component estimates are reduced, compared with the corresponding results from the null model. At these levels, the covariates may be partly explaining homophily (people with similar characteristics tend to be more likely to be connected (McPherson *et al*., 2001)) in the networks with respect to age, sex and ethnic group.

In terms of model fit, the model with all features (the penultimate column) shares the lowest DIC with the model that includes areas and social networks, but not school level (DIC = 2674); this may suggest that the individual characteristics added to these models are to some extent clustered within schools and are thus accounting for some of the school differences. Interestingly, in this example a similar conclusion would have been reached regarding the individual level relationship between academic performance and the three covariates if a single-level regression model had been estimated with the individual level data (the final column), as was found with the more sophisticated models in Table2 that take into account population structure. However, the single-level regression would not allow any potentially important substantive inferences to be made about variation in academic performance at the levels of the population structure above the individual. The fact that models that allow for population structure above the individual level have a statistically better fit suggests that social networks, schools and areas should not be ignored in the analysis.

In Table3, an analysis similar to that of Tables1 and 2 was carried out, but this time the network level was defined as ego-net membership based on the directed adjacency matrix **D**, with the weight matrix calculated as a row-standardized version of **D**. Defining the network by ego-net as opposed to cliques results in slightly better goodness of fit and similar coefficients for the individual level covariates and their standard errors for the model as found, for example, in Table2 for the corresponding models where networks were defined via cliques. The individual level variance component estimate reduces more in the ego-net than for the corresponding clique model, however, and the network variance is higher for the single ego-net component than for either of the clique estimates for the corresponding model in Table2. The school and area level variance components are similar for the full model in Table3 and for the comparable model where the network is defined via cliques (the penultimate column of Table2).

In Table4, the MMMC and network auto-correlation models were compared as closely as possible for models that include only networks and school information. Areas were not considered in the comparison because of their close correspondence to schools, and also because the Adolescent Health Study is a school-based study. The model comparison was achieved as follows. Because the sample is evenly distributed across the schools, nine fixed indicator variables were created for the 10 schools, choosing the first school as the reference group. An MM model was then fitted with networks defined by ego-nets, and an ND model was also estimated, using the same weight information in both cases: the row standardized version of the adjacency matrix. An MM rather than an MMMC model is fitted for this comparison, because only one classification is used in this model (ego-nets), and schools are added as fixed covariates. The network disturbance models were run using the lnam command in the sna package in R, and the results are estimated via maximum likelihood, so that their goodness-of-fit statistic is based on the Akaike information criterion AIC rather than DIC for the MM models that were estimated via Markov chain Monte Carlo sampling though the two measures are comparable. The ND model was compared with the MM model because in both models the network information appears in the random (error) part of the model. A network effects model was not considered because in that case the effect of the social network is modelled directly through the response variable. In Table4 a model was fitted with just a constant and the school indicators. A full model was also specified, that also includes the three individual level covariates. The two models have comparable results in terms of the estimated coefficients, their standard errors and the model goodness of fit.

### 5. Conclusion and discussion

An approach for estimating the nature and extent of social network dependences on individual responses allowing for other group dependences has been demonstrated, using MMMC models. This approach takes into account social network and group dependences when estimating an individual level relationship and allows the variation in individual level responses at classifications of the population structure above the individual level to be compared. The model can be fitted in software for multilevel modelling, such as MLwiN. The model results suggest that network dependences exist, even when other classifications of the population structure such as school and areas are accounted for. A practical approach for estimating the relative share of variation in the individual response at the group and network levels has been proposed, involving the use of average network subgroup membership weights, and this suggests that some of the variation in academic performance can be attributed to social networks.

These new approaches compare well in terms of goodness of fit with existing models used for social network dependences, such as network auto-correlation models. Substantively, there are implications for assuming these models: in particular it is assumed for these models that the social network is exogenous to the individual response. This is a different assumption from those models that allow the coevolution of social networks and behaviour, and hence selection and influence, to be assessed with longitudinal network data: stochastic actor-based models (Snijders *et al*., 2010). The models presented here are not an alternative to stochastic actor-based models; they have a different role. In a descriptive sense, the models presented here allow the variation in individual responses to be assessed for different aspects of the population structure, including social networks. Hence, using these MMMC approaches, it is possible to ask ‘Is there more variation in academic performance between networks, between schools or between areas?’, and to obtain measures of variation before and after the addition of other variables to the model, such as individual characteristics, that might capture homophily in the social networks. This approach could be applied to other situations given the availability of data, e.g. individuals, households, social networks and geographical groups.

Various further extensions could be made to the MMMC model. For example, given the multiple waves of the Adolescent Health Study data, time could be included in the model to allow further inferences about variations in individual responses through time in the context of other population structural features. Also, covariates could be given random coefficients for different classifications. For example, it is possible to make ‘female’ random for cliques or ego-nets to assess whether social network structure has a different association with the response variable for girls from that for boys. The MMMC model could also be applied to a dichotomous response, and other categorical responses could also be modelled, e.g. an ordinal response for self-assessed health status (excellent, good, fair or poor). Another application of these methods is to multiplex networks, where the different types of connection are specified as a series of weight matrices in the MMMC model specification. Moreover, a bivariate response could be considered within the MMMC model framework. Finally, other data could be combined with the network data in the MMMC model framework; for example, census information could be combined with the survey data at area level, if the areas were identifiable in the survey data.

Models that are based on ego-net connections capture direct relationships between people, whereas those that involve cliques explicitly capture higher order substructures in the network. For this Adolescent Health Study example, both models performed similarly in terms of goodness of fit. The decision about how to model the network may depend on the substantive theories to be tested; whether these relate to clusters in the network or simply to connected individuals. The MMMC model framework would allow other network configurations to be included and an important part of the modelling is deciding what social network definitions to include.
